# Using Intensive Longitudinal Data Collected via Mobile Phone to Detect Imminent Lapse in Smokers Undergoing a Scheduled Quit Attempt

**DOI:** 10.2196/jmir.6307

**Published:** 2016-10-17

**Authors:** Michael S Businelle, Ping Ma, Darla E Kendzor, Summer G Frank, David W Wetter, Damon J Vidrine

**Affiliations:** ^1^ Department of Family and Preventive Medicine University of Oklahoma Health Sciences Center Oklahoma City, OK United States; ^2^ Stephenson Cancer Center Oklahoma Tobacco Research Center Oklahoma City, OK United States; ^3^ Children’s Medical Center Division of Population Health Dallas, TX United States; ^4^ Department of Population Health Sciences and the Huntsman Cancer Institute University of Utah Salt Lake City, UT United States

**Keywords:** smartphone, mobile app, mhealth, ecological momentary assessment, smoking cessation, socioeconomic disadvantage, risk estimation

## Abstract

**Background:**

Mobile phone‒based real-time ecological momentary assessments (EMAs) have been used to record health risk behaviors, and antecedents to those behaviors, as they occur in near real time.

**Objective:**

The objective of this study was to determine if intensive longitudinal data, collected via mobile phone, could be used to identify imminent risk for smoking lapse among socioeconomically disadvantaged smokers seeking smoking cessation treatment.

**Methods:**

Participants were recruited into a randomized controlled smoking cessation trial at an urban safety-net hospital tobacco cessation clinic. All participants completed in-person EMAs on mobile phones provided by the study. The presence of six commonly cited lapse risk variables (ie, urge to smoke, stress, recent alcohol consumption, interaction with someone smoking, cessation motivation, and cigarette availability) collected during 2152 prompted or self-initiated postcessation EMAs was examined to determine whether the number of lapse risk factors was greater when lapse was imminent (ie, within 4 hours) than when lapse was not imminent. Various strategies were used to weight variables in efforts to improve the predictive utility of the lapse risk estimator.

**Results:**

Participants (N=92) were mostly female (52/92, 57%), minority (65/92, 71%), 51.9 (SD 7.4) years old, and smoked 18.0 (SD 8.5) cigarettes per day. EMA data indicated significantly higher urges (*P*=.01), stress (*P*=.002), alcohol consumption (*P*<.001), interaction with someone smoking (*P*<.001), and lower cessation motivation (*P*=.03) within 4 hours of the first lapse compared with EMAs collected when lapse was not imminent. Further, the total number of lapse risk factors present within 4 hours of lapse (mean 2.43, SD 1.37) was significantly higher than the number of lapse risk factors present during periods when lapse was not imminent (mean 1.35, SD 1.04), *P*<.001. Overall, 62% (32/52) of all participants who lapsed completed at least one EMA wherein they reported ≥3 lapse risk factors within 4 hours of their first lapse. Differentially weighting lapse risk variables resulted in an improved risk estimator (weighted area=0.76 vs unweighted area=0.72, *P*<.004). Specifically, 80% (42/52) of all participants who lapsed had at least one EMA with a lapse risk score above the cut-off within 4 hours of their first lapse.

**Conclusions:**

Real-time estimation of smoking lapse risk is feasible and may pave the way for development of mobile phone‒based smoking cessation treatments that automatically tailor treatment content in real time based on presence of specific lapse triggers. Interventions that identify risk for lapse and automatically deliver tailored messages or other treatment components in real time could offer effective, low cost, and highly disseminable treatments to individuals who do not have access to other more standard cessation treatments.

## Introduction

Smoking is the leading preventable cause of death and disease in the United States [1], and the prevalence of smoking is much higher in socioeconomically disadvantaged adults (26.3% smoke) than in the general US population (16.8% smoke) [2]. Multiple studies have indicated that smoking cessation interventions are less effective for socioeconomically disadvantaged adults [3-5] despite similar numbers of quit attempts among those with higher and lower socioeconomic status [6,7]. This disparity in treatment effectiveness is likely multicausal. For example, studies have indicated that lower socioeconomic status increases the likelihood of smoking lapse through its effects on increasing stress, nicotine cravings, and other variables [5,8]. In addition, characteristics of socioeconomic disadvantage (eg, lack of insurance, lack of a telephone number or stable address, unreliable transportation, comorbid illnesses) can preclude participation in clinical trials [9,10]. Thus, smoking cessation interventions may not be optimally designed for lower socioeconomic status populations [9,11]. Studies that specifically focus on improving our conceptual models regarding the predictors of smoking lapse and relapse in socioeconomically disadvantaged adults could inform novel treatments for this understudied and underserved population of smokers.

Researchers have developed models for assessing risk for many diseases including breast cancer [12,13], diabetes [14], and cardiovascular disease [15-18]. These risk estimation models often use personal characteristics (eg, family history, age, race or ethnicity), biological variables (eg, lab test results, genetic profile, weight), and current or historical health behaviors (eg, smoking status, heavy alcohol use) to estimate relative risk for particular diseases. These models have proven effective in identifying individuals who should be screened for disease and those who would be most likely to benefit from specific treatments [13-15,17]. Furthermore, risk estimation models have guided medical decision making in systems with limited resources, likely reducing morbidity and mortality. The nearly ubiquitous use of technology in daily life may pave the way toward the development and use of “just-in-time” risk estimation models, including pairing real-time risk estimation with novel behavior change interventions.

To date, most studies that have examined smoking and smoking cessation in socioeconomically disadvantaged smokers have used traditional questionnaire assessment methodology. Study participants typically arrive at a lab or clinic for their baseline visit and are asked to answer questions about their “average” or “recent” (eg, over the past 2 weeks) mood, level of stress, and smoking urges. Participants return to the lab or clinic for follow-up visits and are asked to report thoughts, feelings, and activities that occurred days or even weeks earlier (eg, “How stressed were you when you smoked your first cigarette after your quit date?”). This type of assessment methodology may result in biased or inaccurate estimates due to recall biases and errors in memory [19,20] and offers only a gross understanding of how biopsychosocial variables (eg, withdrawal, stress, craving, alcohol use) effect smoking lapses and relapse. A more nuanced picture of these symptoms may offer important insights that may be used to create or improve cessation interventions for socioeconomically disadvantaged smokers, who face unique and substantial challenges in quitting smoking.

Ecological momentary assessment (EMA) techniques use devices (eg, mobile phones) to repeatedly assess experiences in the natural environment [21]. Thus, EMAs reduce bias and reliance on memory to produce ecologically valid data. Many studies have used EMAs to identify predictors of smoking lapse risk in smokers undergoing a quit attempt. In fact, Schüz et al recently reported that 129 published studies used EMAs to examine smoking in just the past 3 years [22]. Findings from these studies have yielded insights into the lapse and relapse process that can be used to design new, innovative, and more effective smoking cessation interventions. For instance, studies have indicated that sudden stressors are better predictors of smoking lapse compared with more chronic background stress [23], acute and rising negative affect often precedes smoking lapse [24,25], and exposure to other smokers and environmental smoking cues contributes to specific lapse episodes [25]. Additionally, our research team recently showed that trajectories of four variables that were repeatedly measured via mobile phone (ie, negative affect, stress, restlessness, and positive coping expectancy) each predicted confirmed smoking cessation on the quit date in a sample of homeless adults seeking cessation treatment [26].

To date, no studies have used data collected in real time in real-life environments to monitor and assess current risk of smoking lapse, although mobile phone technologies now allow for this type of risk assessment. The development of real-time lapse risk estimators that have high discriminatory accuracy (ie, differentiating moments of high and low lapse risk) could lead to significant improvements in smoking cessation treatments and treatment delivery. For instance, real-time lapse risk assessments could be paired with treatment messages that are tailored to the current situation and needs of the individual and delivered in near real time, when they are most needed. This type of just-in-time adaptive intervention may improve upon tailored treatments, which are more effective than standard nontailored interventions [27], and may usher in the next generation of treatments that are tailored in real time for real-life situations [28,29]. The purpose of the current study was to use EMA data that were collected as part of a clinical trial conducted in a safety-net hospital tobacco cessation clinic to determine if commonly cited smoking lapse risk factors could be combined to create real-time smoking lapse risk estimators.

## Methods

### Participants and Procedure

Data for the current study are from a clinical trial that compared usual tobacco clinic care at a Dallas-based safety-net hospital (usual care [UC]; group counseling and smoking cessation pharmacotherapy) to UC plus small financial incentives for biochemically verified smoking abstinence (contingency management [CM]) [30]. Individuals were eligible to participate in the parent study if they were at least 18 years old, could read English at the 7^th^ grade level or higher [31], smoked at least five cigarettes per day, provided an expired breath sample indicative of smoking (ie, carbon monoxide levels ≥8 parts per million [ppm]), and were willing to quit smoking 1 week after the baseline visit. The parent study randomized 146 participants to UC or CM.

Participant flow through the study is provided in detail elsewhere [30]. Briefly, individuals completed informed consent and were screened for study inclusion. Those who met study inclusion criteria completed in person visits on the day of study enrollment (ie, baseline visit) and each week thereafter for 5 weeks (six visits total). The quit date was scheduled to occur 1 week after the baseline visit. At the baseline visit, participants were instructed on how to use mobile phones provided by the study to complete five automatically prompted EMAs each day for 2 weeks (ie, 1 week pre-quit and 1 week postquit). Specifically, the phone automatically prompted a daily diary assessment by ringing and vibrating 30 minutes after each participant’s self-reported usual waking time, and four additional assessments were prompted each day at random times during normal waking hours (ie, random assessments were prompted roughly every 4 hours). Participants were asked to self-initiate EMAs when they had an urge to smoke and when they were about to lapse. Data collected during the baseline and 1 week postquit visits and during EMAs that were collected during the first week after the scheduled quit date were used for the current study.

This study was approved by the Institutional Review Boards at the University of Texas School of Public Health and University of Texas Southwestern Medical Center. Data collection occurred from August 2011 through June 2013.

### Measures

#### Demographic Characteristics

Participants answered a series of questions during the baseline visit using tablet or laptop computers provided by the study. Participants used headphones to listen to questions that were read aloud by the computer and answered items by using the mouse or tablet touch screen. Questions asked about age, race or ethnicity, sex, current smoking rate, years smoking, income, insurance status, and employment status.

#### Ecological Momentary Assessment Measures

Participants read assessment items that were displayed on the mobile phone screen and touched the screen to select answers to each question. Each EMA assessed current urge to smoke (ie, “I have an urge to smoke”) [26,32], current stress (ie, “I feel stressed”) [26,33], and current cessation motivation (ie, “I am committed to being smoke free”) [26]. Each of these questions required a response on a 5-point Likert-type scale that ranged from strongly disagree to strongly agree. Participants were also asked about current cigarette availability (ie, “Cigarettes are available to me”) with the following answer options: not at all, with extreme difficulty, with difficulty, fairly easily, easily available [25]. Participants responded “yes” or “no” to “Is anyone you are interacting with smoking?” [25,32] and “I drank alcohol within the last hour” [32]. Each of these EMA items have been associated with smoking cessation or lapse.

#### Smoking Status

Smoking status was assessed via EMA every day and in-person on the quit date (1 week after baseline) and 1 week after quit visits. In-person assessments of smoking status were verified using a Vitalograph carbon monoxide monitor. Participants who self-reported abstinence since 10 p.m. on the night prior to their quit date visit and provided a carbon monoxide sample with ≤10 ppm in expired breath were considered abstinent [30,33,34]. Participants who self-reported abstinence (ie, not smoking even a puff) since the quit date and provided a sample with <8 ppm at the 1 week postquit follow-up visit were considered abstinent. Participants who reported smoking cigarettes on any EMA during the postquit week but reported continuous abstinence since their quit date during in-person assessments were excluded from the current analyses.

### Development of Lapse Risk Estimators and Statistical Analyses

The smoking lapse risk estimator was developed using a multistep process. First, for all participants who lapsed, the time and date of the first lapse were marked in the dataset. Second, all postquit EMAs collected prior to the first reported lapse were selected and retained in the dataset. All postquit EMAs for those who did not lapse were retained in the dataset. Third, the number of lapse risk factors present during each EMA was calculated to create a lapse risk score (ie, agreeing/strongly agreeing to the presence of smoking urges and feeling stressed each received 1 point, disagreeing/strongly disagreeing to a commitment to being smoke free received 1 point, endorsing fairly easily/easily available cigarettes received 1 point, interacting with someone who was smoking received 1 point, and consuming alcohol in the past hour received 1 point). Thus, the EMA-derived lapse risk score could range from 0 to 6 points. Fourth, lapse risk scores during EMAs that occurred within 4 hours prior to lapse for those who lapsed were compared to lapse risk scores for all other EMAs (ie, EMAs for those who did not lapse in the first postquit week and EMAs that were collected prior to the specified lapse time period) to determine if lapse risk scores were symptomatic of imminent lapse. Fifth, with consideration that some variables may have a larger impact on lapse than other variables, various techniques for weighting the lapse risk variables were examined to determine if the sensitivity and specificity of the unweighted lapse risk estimator could be improved. For example, iterative strategies examined the effects of applying various weights (eg, 0.1, 0.2, 0.3) to each lapse risk factor on the sensitivity and specificity of the risk estimator [18,35]. The area under the curve in the weighted and unweighted estimators was compared.

Demographic variables and EMA measures were summarized using the mean and standard deviation for continuous variables and frequency for categorical variables. The proximity of each EMA measure to the first lapse was identified. EMAs were categorized as occurring (1) within 4 hours of the first lapse, (2) more than 4 hours before the first lapse in those who lapsed during the first week after cessation, or (3) at any time for individuals who did not lapse during the first week after cessation. Mixed-effects regression analyses were conducted to identify differences in EMA-assessed risk factors (eg, urge, stress, low cessation motivation, cigarette availability, alcohol use, interaction with others smoking) and total number of lapse risk factors between the three groups defined by proximity to first lapse, accounting for treatment group (α=.05). Data were analyzed using STATA 13.0 (STATA Corp).

## Results

Data from 92 participants were included in the current study. Specifically, participants consisted of 52 adults who identified the moment of their first smoking lapse during the first week of a smoking cessation attempt and 40 participants who maintained verified abstinence throughout the first postquit week. The remaining study participants (ie, 54 adults) were not included in the current study because the moment of their first smoking lapse could not be determined or the participant provided inconsistent information about abstinence (ie, EMA and in-person assessments of abstinence were inconsistent or carbon monoxide measurements did not support self-reported abstinence status).

Participants (N=92) were mostly female (57%, 52/92), African American or other racial or ethnic minority (71%, 65/92), and 51.9 years old (SD 7.4) on average. Most participants were socioeconomically disadvantaged: 88% (81/92) had annual household incomes below US $25,000, 54% (50/92) were uninsured, and 82% (75/92) were unemployed. Participants smoked 18.0 cigarettes (SD 8.5) per day and had been smoking for 30.1 years (SD 9.2) on average. Participants completed a total of 4005 EMAs (mean 43.5 EMAs per participant) during the 7-day postquit period. The total number of EMAs completed by lapsers (n=52) and nonlapsers (n=40) during the first postquit week did not differ (*P*=.64). In total, 108 assessments were completed within 4 hours of the first smoking lapse, lapsers completed 322 assessments more than 4 hours before the first lapse, and 1722 assessments were completed by participants who did not lapse during the first postquit week. This subset of 2152 EMAs were included in the analyses. Because the primary aim was to use EMA data to estimate imminent risk for initial smoking lapse, the 1833 EMAs that were collected after the first lapse were not included in the current analyses.

EMA data indicated significantly higher urges (*P=*.01), stress (*P=*.002), alcohol consumption (*P*<.001), interaction with someone smoking (*P*<.001), and lower cessation motivation (*P*=.03) within 4 hours of the first lapse compared with EMAs collected when lapse was not imminent. Further, the total number of lapse risk factors present within 4 hours of lapse (mean 2.43, SD 1.37) was significantly higher than the number of lapse risk factors present during periods when lapse was not imminent (mean 1.35, SD 1.04, *P*<.001). See Table 1 for the prevalence of lapse risk factors and differences between risk factors assessed during EMAs collected within 4 hours of lapse and when lapse was not imminent.

**Table 1:** EMA-assessed risk factors by proximity to first lapse (analyses controlled for treatment group).

	Lapsers, %		Abstainers, % n=1722
	Within 4 hours of first lapse n=108	˃4 hours before first lapse n=322	
Urge	59.3^a^	49.1^c^	32.8^a,c^
Stress	41.1^b^	18.8^b^	25.9
Low cessation motivation	17.3^a^	15.1^c^	1.0^a,c^
Cigarette availability	74.8^a^	70.4	52.6^a^
Alcohol use	19.1^a,b^	18.9^b,c^	3.4^a,c^
Interacting with others smoking	33.6^a,b^	12.9^b^	12.1^a^
Number of lapse risk factors	2.43^a,b^	1.83^b,c^	1.27^a,c^

^a^Risk factors different (*P*<.05) in EMAs collected ≤4 hours of first lapse and abstainers.

^b^Risk factors different (*P*<.05) in EMAs collected ≤4 and >4 hours of first lapse.

^c^Risk factors different (*P*<.05) in EMAs collected >4 hours of first lapse and abstainers.

**Figure 1:**
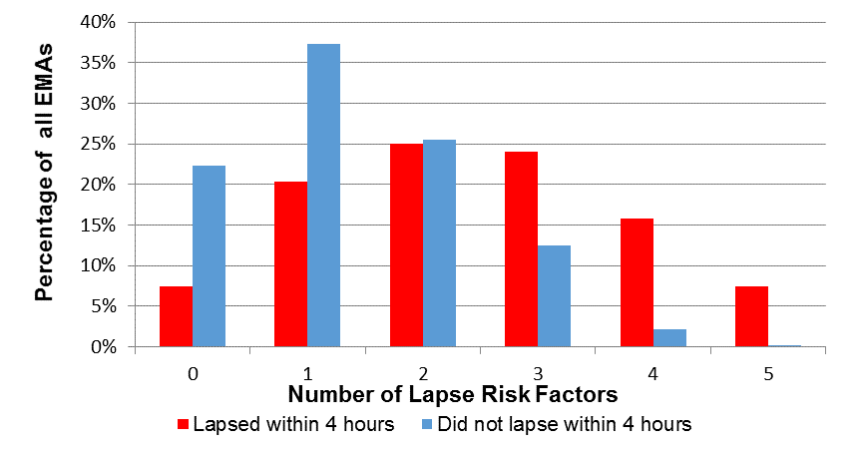
Number of lapse risk factors by imminent lapse status.

**Figure 2:**
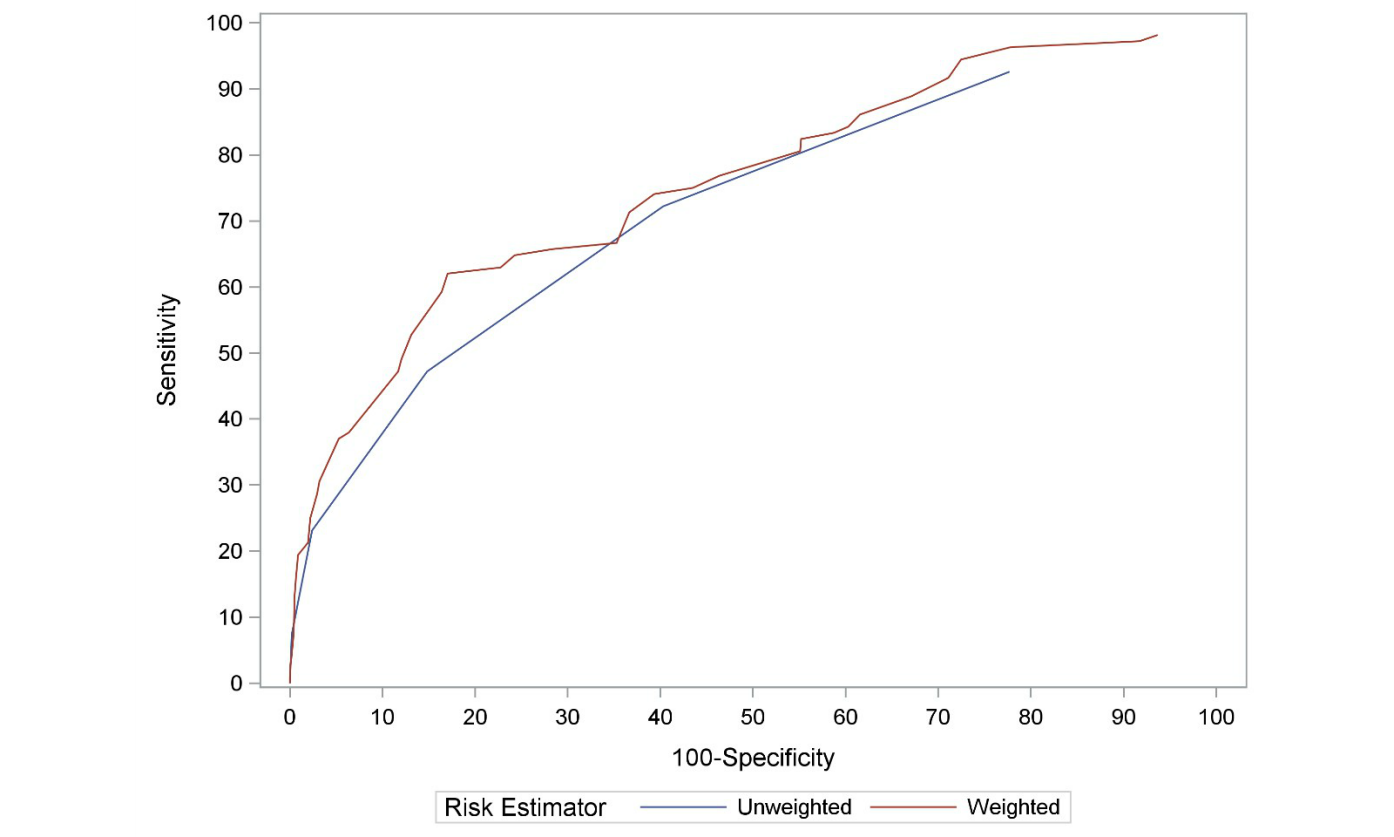
Receiver operator characteristics curve for weighted and unweighted risk estimators.

As indicated in Figure 1, imminent lapse was much more common when participants endorsed at least 3 lapse risk factors. Specifically, lapsers endorsed ≥3 lapse risk factors in 47.2% (51/108) of EMAs completed within 4 hours of the first lapse. Participants who did not lapse during the first week of their cessation attempt endorsed ≥3 lapse risk factors in only 11.90% (205/1722) of all postquit EMAs (see Figure 1). Using a cut-off score of 3, the lapse risk estimator correctly identified imminent lapse in 47.2% (51/108) of all EMAs collected within 4 hours of lapse and correctly classified 85.18% (1741/2044) of all EMAs where lapse was not imminent. Importantly, 62% (32/52) of all participants who lapsed completed at least one EMA where they reported ≥3 lapse risk factors within 4 hours of their first lapse. The receiver operator characteristics (ROC) curve in Figure 2 indicates the sensitivity and specificity of the unweighted lapse risk estimator.

Various variable weighting strategies were examined to determine if weighting variables could improve the predictive ability of the lapse risk estimator. We settled on a strategy that weighted some variables more heavily than others and allowed variables to indicate increased or decreased risk of lapse. Specifically, we found that the best weighting (ie, maximizing sensitivity and specificity for the overall risk estimator) for “I have an urge to smoke” (response options ranged from 5=strongly agree, 3=neutral, and 1=strongly disagree) was to subtract 3 and multiply by 0.2 (ie, the effect of smoking urge on lapse was much smaller than some other variables included in the lapse risk estimator). This weighting allowed for low urge ratings to indicate reduced risk for lapse and high urge ratings to indicate heightened risk for lapse. The stress and cessation motivation items were weighted in a similar manner. However, interacting with other smokers and recent alcohol consumption received full points in the final lapse risk estimator formula. Interestingly, recent alcohol consumption, while much less frequently endorsed, had a much larger impact on smoking lapse risk. Finally, the best weighting of the cigarette availability item was to subtract 3 (ie, 3=“with difficulty”) and multiply by 0.7. The final weighted EMA lapse risk estimator formula is as follows:

Lapse risk score = (urge ‒ 3) x 0.2 + (stress ‒ 3) x 0.2 + (cigarette availability ‒ 3) x 0.7 + (interacting with someone smoking [yes =1; no=0]) + (recent alcohol use [yes=1; no=0]) ‒ (cessation motivation ‒ 3) x 0.2

**Figure 3:**
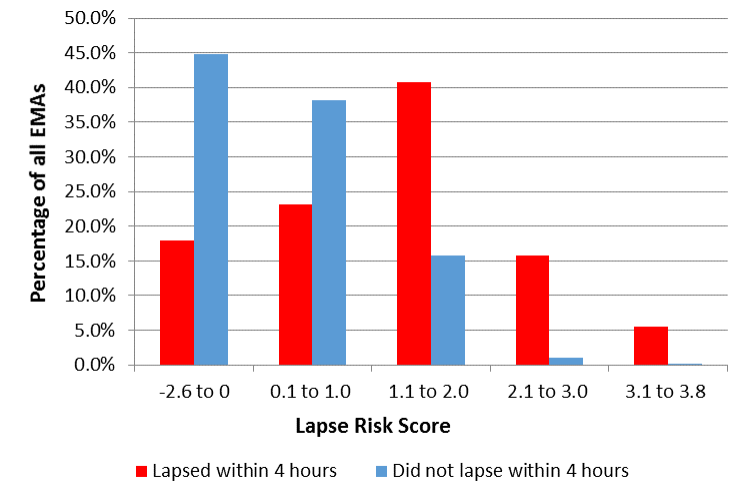
Weighted lapse risk scores by lapse status.

Scores on the six-variable weighted lapse risk estimator could range from -2.6 to 4.2. As shown in Figure 3, imminent lapse was much more common when the weighted lapse risk score was greater than 1.0. Using a lapse risk cut-off score of 1.0, 62.0% (67/108) of all EMAs collected within 4 hours of a lapse were indicative of imminent lapse. Among EMAs in which lapse did not occur within 4 hours of the assessment, 16.98% (347/2044) were above the lapse risk cut-off score (see Figure 3). Thus, the weighted lapse risk estimator had a sensitivity of 62.0% and a specificity of 83.0%. Importantly, 80% (42/52) of all participants who lapsed had at least one EMA with a lapse risk score above the cut-off within 4 hours of their first lapse. The ROC displayed in Figure 2 indicates the sensitivity and specificity of the weighted lapse risk estimator. Analysis indicated that the area under the curve was larger in the weighted (area=0.76, 95% CI=0.71-0.81) compared to the unweighted (area=0.72, 95% CI=0.67-0.77) estimator (*P*<.004).

## Discussion

### Principal Findings

The current study used mobile phone‒based EMA data to estimate risk of imminent smoking lapse in a sample of smokers seeking cessation treatment at a safety-net hospital clinic. Six commonly cited smoking lapse risk factors were collected multiple times each day and used to assess risk for imminent (ie, within the next 4 hours) smoking lapse. Study results yielded three key findings. First, lapse risk estimation using real-time mobile phone‒based momentary assessments is feasible in socioeconomically disadvantaged smokers seeking cessation treatment. In fact, unweighted and weighted lapse risk estimators distinguished the majority of all lapsers within 4 hours of the first lapse. The presence of three or more lapse risk factors during momentary assessments was indicative of imminent lapse (ie, within 4 hours) in 62% of all lapsers during the first week of a scheduled quit attempt. However, the presence of three or more lapse risk factors did not always correspond to imminent lapse (ie, this was the case for 15% of all EMAs where lapse was not imminent). Second, differential weighting of lapse risk factors improved the lapse risk estimator. Specifically, the weighted lapse risk estimator identified 80% of all first lapses within 4 hours of the lapse while retaining a relatively low rate of false positives (ie, 17% false positive rate; 83% of true negatives were correctly identified as low risk for imminent lapse). Although choosing a lower lapse risk cut-off score would have increased the number of EMAs that were correctly identified as high risk for imminent lapse, the cost would be a greater proportion of false positives (ie, prediction of lapse when no lapse actually occurs; see ROCs in Figure 2). A third key study finding is that many participants were able to successfully cope with multiple lapse risk factors without lapsing. However, maintaining smoking abstinence in the presence of three or more of the identified lapse risk triggers was rare. Further examination of situations where participants successfully coped with heightened lapse risk is warranted and will be the focus of future analyses.

Across a range of health behaviors, tailored treatments are typically superior to the more commonly used “one-size-fits-all” treatment approach [36-38]. Treatment tailoring typically uses participant characteristics that are assessed at the baseline visit (eg, gender, level of dependence). Balmford and Borland recently used participant quitting stage (pre-quit, setting a quit date, around the quit date, and lapse) to tailor a text messaging smoking cessation intervention [39]. They also tailored the intervention to age, nicotine dependence, and gender. Most participants reported that this intervention was helpful (ie, 87.1%), and participants were willing to receive messages over long periods (ie, two thirds of participants received messages for 20-35 days) [39]. Future interventions may take this approach a step further through the use of dynamic tailoring, that is, tailoring based on data that are collected during successive EMAs. More specifically, tailored smoking cessation treatment messages (eg, text-and video-based treatment messages) may be delivered based on current lapse risk and currently present lapse risk factors (eg, stress, alcohol use, smoking urge) in real time in the natural environment.

The potential for EMA-informed treatments has only recently become possible due to the substantial increase in mobile phone ownership and use. Most Americans (ie, 72% in 2015) have active smartphones and the smartphone market share is rapidly increasing among socioeconomically disadvantaged populations [40]. For example, 50% of those who earned <US $30,000 per year reported active smartphones in 2015 [41]. Thus, mobile phone‒based smoking cessation apps that continuously assess for smoking lapse risk in near real time and automatically intervene may increase the ability to reach and intervene with socioeconomically disadvantaged smokers—a population with substantial barriers that hamper use of traditional smoking cessation treatments [42].

### Limitations

Study findings should be considered with limitations. First, the sample was small, mostly African American, and impoverished; thus, results may not generalize to nonminority and higher income smokers. Second, many (n=54) individuals who participated in the parent study were excluded from the current analyses because the exact moment of lapse could not be determined or self-reported, and biologically confirmed abstinence was inconsistent. Identification of the moment of smoking lapse requires participant vigilance and is vulnerable to bias. Future studies should develop more passive ways to detect smoking lapse. For instance, wearable devices may be used to detect breathing patterns [43] or hand and arm gestures [44,45] that are suggestive of smoking. Third, participants were followed with EMA only during the first week after cessation, thus, the utility of the risk estimator beyond the first week after cessation is unknown. Fourth, participants received compensation for completing EMAs that were prompted by the mobile phone. Future research is needed to determine if smokers who are undergoing a smoking cessation attempt will complete brief EMAs multiple times per day without incentives. Fifth, weighting of risk estimation items was based on examination of the study data and may have resulted in overfitting the data. Unfortunately, the sample size was not large enough to conduct cross-validation analyses. Study findings should be replicated prior to use of this lapse risk estimator in other populations.

### Conclusion

Real-time smoking lapse risk estimation is feasible in socioeconomically disadvantaged individuals seeking smoking cessation services. This type of lapse risk estimator may be used to estimate the likelihood of smoking lapses in near real time, enabling the creation of interventions that utilize EMA data to prompt tailored interventions that address patient needs in real time. Interventions that identify risk for lapse and automatically deliver tailored messages or other treatment components in real time could offer effective, low cost, and highly disseminable treatments to individuals without access to other more standard cessation treatments.

## Abbreviations

EMA: ecological momentary assessment
ROC: receiver operator characteristics
